# Dose-Dependent Anti-Inflammatory Effects of Live and Heat-Treated *Ligilactobacillus salivarius* and *Bifidobacterium breve* via NF-κB and COX-2 Modulation in an In Vitro Model of Airway Inflammation

**DOI:** 10.3390/nu17152504

**Published:** 2025-07-30

**Authors:** Marta Pagnini, Annalisa Visciglia, Giovanni Deusebio, Marco Pane, Alessandro Celi, Angela Amoruso, Tommaso Neri

**Affiliations:** 1 Dipartimento di Patologia Chirurgica, Medica, Molecolare e dell’Area Critica, University of Pisa, 56126 Pisa, Italy; marta.pagnini@med.unipi.it (M.P.); alessandro.celi@unipi.it (A.C.); 2 Centro Dipartimentale di Biologia Cellulare Cardiorespiratoria, University of Pisa, 56126 Pisa, Italy; 3 Research and Development, Probiotical Research, 28100 Novara, Italy; a.visciglia@probiotical.com (A.V.); g.deusebio@probiotical.com (G.D.); m.pane@probiotical.com (M.P.); a.amoruso@probiotical.com (A.A.)

**Keywords:** lung inflammation, pulmonary epithelial cells, probiotics, postbiotic, *Ligilactobacillus salivarius*, *Bifidobacterium breve*, anti-inflammatory effect, cytokine modulation

## Abstract

Background: Probiotics are live microorganisms known for their health-promoting effects, particularly in modulating immune responses and reducing inflammation within the gastrointestinal tract. Emerging evidence suggests probiotics may also influence respiratory health, prompting investigation into their potential therapeutic application in lung inflammation. Methods: This study examined the anti-inflammatory effects of *Ligilactobacillus salivarius* (LS01 DSM 22775) and *Bifidobacterium breve* (B632 DSM 24706) on inflamed pulmonary epithelial cells. Lung carcinoma epithelial cells (A549) and normal bronchial epithelial cells (16HBE) were stimulated with IL-1β and treated with viable and heat-treated probiotics. Results: CCL-2 levels were significantly reduced by up to 40%, in A549 by viable form (10^5^–10^7^ AFU/g), instead of in 16HBE by heat-treated form (10^7^–10^9^ TFU/g). In A549 cells, TNF-α decreased by 20–80% with all formulations; instead, in 16HBE cells, IL-8 was reduced by viable strains (10^7^ AFU/g) by approximately 50%, while heat-treated strains (10^9^ TFU/g) decreased both IL-6 and IL-8 by 50%. All effective treatments completely inhibited IL-4 and eotaxin and suppressed NF-κB activation in both cell lines, with up to 80% reduction in phospho-p65 levels. In A549 cells, heat-treated strains fully blocked PGE2 production; instead, all four probiotics significantly inhibited COX-2 expression by approximately 50%. Conclusions: These findings demonstrate that both viable and heat-treated probiotics can modulate inflammatory responses in pulmonary epithelial cells, suggesting their potential application in inflammatory respiratory diseases. Heat-treated formulations may be particularly suited for local administration via inhalation, offering a promising strategy for targeting airway inflammation directly.

## 1. Introduction

Airway inflammation plays a central role in the pathogenesis of a wide range of respiratory disorders, including asthma, chronic obstructive pulmonary disease (COPD), cystic fibrosis and respiratory infection. These conditions are characterized by aberrant immune responses, epithelial barrier dysfunction and microbial dysbiosis, which collectively contribute to disease onset, progression and exacerbation [1,2]. The lung, once considered a sterile organ, is now recognized as harboring a unique microbiome that, although less dense than that of the gut, plays a crucial role in immune homeostasis and respiratory health [3]. Culture-independent microbial profiling has revealed microbial DNA in the lungs of healthy individuals, highlighting the presence of resident microbial communities [4]. These microbial communities originate primarily from the oral and nasal microbiota via microaspiration [3] and differ in composition from gut microbiota despite phylogenetic similarities [5]. Changes in the lung microbiota have been associated with chronic inflammatory respiratory diseases, supporting the hypothesis that the lung microbiota influence both respiratory health and disease [5]. Increasing evidence highlights the existence of bidirectional communication between the gut and the lungs, referred to as the gut–lung axis. This axis is rooted in the common embryological origin of the gastrointestinal and respiratory tracts, their shared mucosal immune system and the ability of microbial metabolites or components to influence distal mucosal sites [4]. Dysbiosis of the gut microbiota, whether caused by infection or antibiotic use, can lead to systemic immune dysregulation and has been implicated in the pathophysiology of respiratory diseases [6,7]. This highlights the importance of a balanced microbial community for maintaining immune homeostasis and lung health. Among the strategies aimed at restoring microbial balance and modulating inflammation, probiotics have gathered increasing attention. In 2001, an expert consultation by FAO and WHO defined probiotics as “live microorganisms which, when administered in adequate amounts, confer a health benefit on the host” [8]. Probiotics play multiple roles and exert their effects through various mechanisms, one of the most important being the modulation of the host’s microbiota [9]. They have several functions in the human body, with one of their primary benefits being the ability to colonize the host and help to maintain a balance between pathogens and beneficial bacteria that are necessary for normal human body functioning [10]. A crucial mechanism of probiotic action is immune modulation, particularly through the regulation of inflammatory signaling pathways. Probiotics can influence both local and systemic immune responses by releasing cell wall fragments or DNA into the intestinal lumen [9,11]. They interact directly with the immune system, balancing pro- and anti-inflammatory cytokines [12]. Specifically, probiotics have been shown to downregulate the nuclear factor kappa-light-chain-enhancer of activated B cells (NF-κB) pathways, a central regulator of inflammation, thereby reducing the expression of pro-inflammatory cytokines such as interleukin (IL)-8, tumor necrosis factor (TNF)-α and IFN-γ and inducing anti-inflammatory mediators like IL-10 and TGF-β [11]**.** For example, probiotic *Lactobacillus* and *Bifidobacterium* strains have been shown to downregulate interleukin (IL)-6 and TNF-α in intestinal epithelial cells, and similar effects have been observed beyond the gut [13,14]. Moreover, in the context of airway inflammation, a key downstream effector of immune activation is the inducible enzyme cyclooxygenase-2 (COX-2), which catalyzes the synthesis of prostaglandins from arachidonic acid. Among these, prostaglandin E2 (PGE2) plays a complex role as both a pro-inflammatory and immunoregulatory mediator. PGE2 is known to modulate dendritic cells and T cell function, influence epithelial barrier integrity and promote mucus secretion in the respiratory tract [15]. Elevated COX-2 expression and PGE2 production have been observed in various inflammatory lung conditions, including asthma and COPD, contributing to the chronicity of inflammation and tissue remodeling [16,17]. Interestingly, certain probiotic and probiotic-derived products have shown the capacity to modulate COX-2 and PGE2 release, suggesting a potential mechanism through which microbial intervention may attenuate pulmonary inflammation [18,19]. This highlights the importance of targeting not only cytokines signaling but also lipid mediators in the broader context of immunomodulatory strategies. Although most researchers have focused on gastrointestinal effects, probiotics have also shown promise in respiratory contexts. In children with asthma, a heterogeneous disease characterized by chronic airways inflammation, reversible airflow limitation and bronchial hyperreactivity, probiotics, alone or in a mixture, have been reported to reduce exacerbation, improve lung function, alleviate respiratory symptoms and attenuate lung inflammation [20,21,22,23]. However, certain applications present inherent formulation and stability challenges that limit the use of viable microorganisms. In particular, for pulmonary delivery routes, the administration of live strains raises specific safety concerns due to the potential risk of respiratory colonization and infection in immunocompromised patients [24,25,26,27]. Additionally, maintaining the viability of probiotics in pharmaceutical formulations such as inhalers, nebulizers, or dry powder formulations poses significant technical hurdles including moisture sensitivity, temperature stability during storag and survival through aerosolization processes [28].

Due to these formulation constraints and route-specific safety considerations, there has been increased interest in the use of non-viable, heat-treated probiotics [29], called postbiotics, which the International Scientific Association of Probiotics and Prebiotics (ISAPP) defines as a “preparation of inanimate microorganisms and/or their constituents that confers a health benefit on the host” [30]. These preparations offer distinct advantages for challenging delivery systems where maintaining microbial viability would be technically infeasible or clinically inadvisable [31].

They can be derived from a well-defined microorganism or combination of microorganisms for which genomic sequences are known and produced by a defined technological process of biomass production and inactivation that can be reliably reproduced. There is considerable published evidence that preparations containing dead cells and their metabolites can induce relevant biological responses, restoring the normal intestinal homeostasis, often in ways similar to those observed with live cells, although with potential differences. In fact, after bacterial inactivation, mainly through heat treatment, dead cells can release bacterial components with important immunomodulatory effects and antagonistic activity against pathogens [29].

With this in mind, the aim of this work was to investigate the potential anti-inflammatory effects of two probiotics strains, in both viable and heat-treated forms, using an in vitro model of lung inflammation.

## 2. Materials and Methods

### 2.1. Probiotics

The probiotic strains *Ligilactobacillus salivarius* LS01 (DSM 22775) and *Bifidobacterium breve* B632 (DSM 24706) were provided by Probiotical Research S.r.l. (Novara-Italy) in both viable and heat-treated (HT) forms.

Viable strains were stored at −80 °C, thawed prior to use and cultured in de Man, Rogosa and Sharpe (MRS) broth; cysteine (0.05% *w*/*v*) was added to the medium for *B. breve* to reduce oxygen levels. After overnight incubation at 37 °C, the cultures were lyophilized and then quantified by flow cytometry using thiazole orange (TO) and propidium iodide (PI) staining to determine active fluorescent units (AFU), which represent metabolically active or membrane-intact cells. The lyophilized biomass was subsequently blended with maltodextrin and standardized to a final concentration of 10^9^ AFU/g.

For postbiotic (heat-treated, HT) formulations, bacterial suspensions were thermally inactivated at temperatures above 75 °C for 30–90 min. The inactivated cells were then spray-dried and quantified by flow cytometry (TO/PI) to determine total fluorescent units (TFU), which include all fluorescent cells detected, both viable and non-viable (damaged or dead), representing the total bacterial cell count regardless of metabolic activity. Final powders were standardized to 10^10^ TFU/g using maltodextrin and stored at 4 °C, along with the viable strain powders, until use in lung model experiments.

### 2.2. Materials

RPMI 1640 medium (catalog #R6504), DMEM (catalog #M5650), HEPES solution (catalog #H3537), penicillin/streptomycin (catalog #P4333), L-glutamine (catalog #G7513), Phosphate buffered saline (PBS) without calcium/magnesium (catalog #P2272), foetal bovine serum (FBS) (catalog #F7524), 3-(4,5-dimethylthiazol-2-yl)-2,5-diphenyltetrazolium bromide (MTT) (catalog #M5655), dimethyl sulphoxide (DMSO) (catalog #472301-1L), protease (catalog #P8340), phosphatase inhibitors (catalog #PHOSS-RO) and dexamethasone (catalog #D4902) were obtained from Sigma-Aldrich (Milan, Italy). Phosphate buffered saline (PBS) with calcium/magnesium (catalog #SKU:P1407-0121) was purchased from D.B.A (Milan, Italy), and Human IL-1β (catalog #10139-HNAE) was purchased from Sino Biological (Dusseldorf, Germany).

### 2.3. Cell Line and Culture Medium

Human alveolar epithelial cells (A549) (American Type Culture Collection, CCL-195) were kindly provided by Dr. R. Danesi (University of Pisa, Pisa, Italy) and maintained in RPMI-1640 supplemented with 10% (*vol*/*vol*) fetal bovine serum (FBS), 0.2 mg/mL L-glutamine, 100 U/mL penicillin and 100 microg/mL streptomycin.

Immortalized bronchial epithelial cells (16HBE) (American Type Culture Collection, CRL-2741) were kindly provided by Dr. M. Profita (National Research Council, Palermo, Italy) and maintained in Dulbecco’s modified Eagle’s medium (DMEM) supplemented with 10% (*vol*/*vol*) FBS, 0.2 mg/mL L-glutamine, 2.5 mM HEPES buffer, 100 U/mL penicillin and 100 microg/mL streptomycin. Both cell lines were maintained in a humidified 95% air/5% CO_2_ atmosphere at 37 °C.

### 2.4. Inflammation Induction and Use of Probiotics

Inflammation was induced in pulmonary epithelial cells using a pro-inflammatory stimulus, IL-1β, the concentration and induction time of which were chosen on the basis of the literature.

Indeed, studies show that stimulation with IL-1β 10 ng/mL for 24 h could be the best solution to simulate an inflammatory condition in vitro [32,33].

Probiotics are supplied by Probiotical Research in powder form. In total, 1 g of a sample is resuspended with sterile water to reach a final volume of 10 mL. Serial dilutions were made to obtain the desired concentration, using antibiotic-free culture medium.

The probiotics were assessed at three different concentrations to investigate their potential anti-inflammatory effect:

*L. salivarius_*V -> Pb1 [10^5^, 10^6^, 10^7^]

*L. salivarius*_HT -> Pb2 [10^7^, 10^8^, 10^9^]

*B. breve_*V -> Pb3 [10^5^, 10^6^, 10^7^]

*B. breve_*HT -> Pb4 [10^7^, 10^8^, 10^9^]

The concentrations used in this study were selected based on regulatory and literature-based evidence. For the viable probiotic, a range of 10^5^–10^7^ AFU/g was tested, centering around 10^6^ AFU/mL, which corresponds to the commonly accepted in vitro equivalent of 10^9^ AFU/day in vivo, as indicated by FDA guidance on translational dosing (https://www.fda.gov/media/72309/download (accessed on 3 June 2025)). For the postbiotic, a range of 10^7^–10^9^ TFU/g was chosen, reflecting the concentrations most frequently reported in the literature for heat-treated probiotics and postbiotic fractions. Examples include Liu, Y.W. et al. in PLoS ONE—2014 [34] and in Beneficial Microbes—2015 [35], which demonstrated immunomodulatory effects within this range. This design ensures both translational relevance and comparability with existing data.

### 2.5. Cell Viability Assay

MTT assays were performed to assess cell viability and to evaluate the cytotoxicity of the probiotics for inflamed cells.

Cells (30,000/well) were stimulated with IL-1β (10 ng/mL) in the presence or absence of probiotics for 24 h. To ensure the complete removal of probiotics from the system, the cells were washed with PBS. MTT (3-(4,5-dimethylthiazol-2-yl)-2,5-diphenyltetrazolium bromide) (100 µL at 5 mg/mL) was added. The cells were incubated at 37 °C for 3 h and then formazan crystals formed by MTT metabolism were solubilized by adding 150 µL of dimethyl sulphoxide (DMSO) to each well.

Absorbance was measured at a wavelength of 570 nm with background subtraction at 630–690 nm using a microplate reader (iMarkTM Microplate Absorbance Reader, Bio-Rad, Milan, Italy) [36].

### 2.6. ELISAs for Inflammatory Cytokines

After stimulation with IL-1β in the presence or absence of probiotics, supernatants were collected, centrifuged at 16.1× *g* for 15 min at 4 °C and used to assess the possible anti-inflammatory effects of the probiotics.

The expression of C-C motif chemokine ligand-2 (CCL-2), tumor necrosis factor α (TNF-α), interleukin-4 (IL-4), interleukin-6 (IL-6), interleukin-8 (IL-8) (Sino Biological, Dusseldorf, Germany) and Eotaxin (RayBiotech, Prodotti Gianni S.p.A, Milan, Italy) were measured by a sandwich ELISA kit according to the manufacturer’s instructions.

Dexamethasone (DEX, Sigma-Aldrich (Milan, Italy), an anti-inflammatory agent that has been shown to reduce levels of several inflammatory cytokines in pulmonary cells where inflammation is induced by IL-1β [37,38,39], was used as a control.

### 2.7. NF-κB Pathway Evaluation

The NF-κB signaling pathway plays a significant role in immune and inflammatory responses, because the nuclear translocation of the NF-κB p65 subunit stimulates the transcription of several proinflammatory genes [40]. NF-κB is composed of two subunits, p50 and p65; in uninduced cells, it remains in the cytoplasm bound to inhibitory proteins IκBs (inhibitors of NF-κB). When cells are stimulated, IκB proteins are phosphorylated, ubiquitinated and degraded. NF-κB can translocate to the nucleus and the p65 subunit can be phosphorylated, allowing it to activate the transcriptional activity [41].

After 15 min of stimulation with IL-1β (10 ng/mL) in the presence or absence of probiotics, supernatants were removed, and cells were treated with lysis buffer (500 µL each 10^6^ cells) supplemented with protease and phosphatase inhibitors and then scraped and centrifuged at 13.000 rpm for 10 min at 4 °C.

After centrifugation, the pellet consisting of cell membranes was discarded; supernatants, containing cell lysates, i.e., nuclear proteins, were collected. Before the detection of phospho NF-κB p65 levels by a sandwich ELISA (RayBiotech, Prodotti Gianni S.p.A, Milan, Italy) according to the manufacturer’s instructions, a protein assay (Bradford assay) was performed to equalize the concentration of proteins loaded into the wells.

### 2.8. COX-2 and PGE2 Pathway Evaluation

Cyclooxygenase 2 (COX-2) is one of the two regulatory enzymes of prostaglandin E2 (PGE2), which can increase inflammation, fever and pain. This isoform is transcriptionally activated and overexpressed in response to many proinflammatory stimuli like IL-1β, IFN-γ and TNF-α and also pathogen stimuli such as lipopolysaccharides.

It breaks down arachidonic acid to release prostaglandins, the free arachidonic acid in the cell cytosol obtained upon the degradation of membrane phospholipids into prostaglandin H2 (PGH2), an active precursor for the synthesis of various prostanoids [42,43]. Subsequently, cell-specific isomerases and reductases result in the production of biologically relevant PGs [44]. After 24 h of stimulation with IL-1β (10 ng/mL) in the presence or absence of probiotics, supernatants were collected for the assessment of PGE2 levels, and cells were treated with lysis buffer (150 µL each 6 × 10^5^ cells) supplemented with protease and phosphatase inhibitors and after 10 min scraped. The plate was kept on ice for 10 min and then centrifuged at 13.000 rpm for 10 min at 4 °C.

After centrifugation, the pellet consisting of cell membranes was discarded; supernatants, containing cell lysates, i.e., nuclear proteins, were collected. Before the detection of COX-2 levels by a sandwich ELISA (BT LAB, Prodotti Gianni S.p.A, Milan, Italy) according to the manufacturer’s instructions, a protein assay (Bradford assay) was performed to equalize the concentration of proteins loaded into the wells.

### 2.9. Statistical Analysis

Data are presented as the mean ± SEM. Comparisons between groups were made by either one-way ANOVA for repeated measurements followed by Bonferroni’s analysis or Student’s paired two-tailed *t*-test, as appropriate. Data were analyzed using Prism Software Version 10.4.1 (GraphPad, San Diego, CA, USA). One-tail values of *p* < 0.05 were considered statistically significant.

## 3. Results

### 3.1. Effects of Probiotics on Cell Viability

In order to assess the possible cytotoxicity of probiotics for IL-1β-induced inflamed A549 and 16HBE cells, they were treated with IL-1β (10 ng/mL) in the presence or absence of probiotics for a period of 24 h.

**Figure 1:**
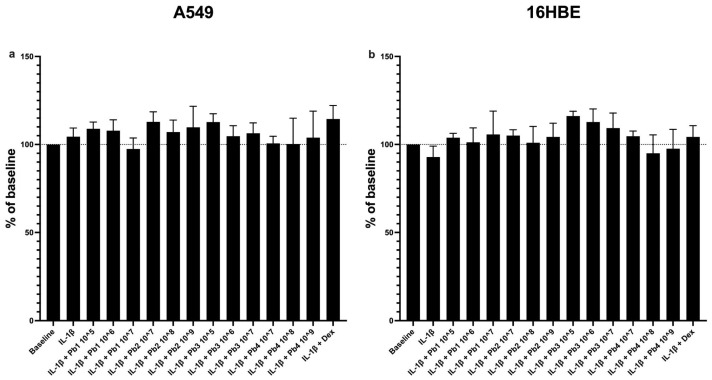
**Effects of probiotics on cell viability in IL-1β-stimulated epithelial cells analyzed using the MTT assay.** A549 (**a**) and 16HBE (**b**). No significant cytotoxic effects were observed upon probiotic treatment in either cell line. Each value represents the mean ± S.E.M. (n = 6 for each panel). The concentration of probiotics used is expressed in AFU/g for viable forms and in TFU/g for postbiotic ones. *L. salivarius*_V: Pb1; *L. salivarius*_HT: Pb2; *B. breve*_V: Pb3; *B. breve*_HT: Pb4.

Cell viability was assessed by means of an MTT assay, which revealed no evidence of probiotic-induced cytotoxicity in either A549 or 16HBE cell lines (Figure 1).

### 3.2. Dose-Dependent Inhibition of the Proinflammatory Cytokines CCL-2 and TNF-α by Probiotics on IL-1β-Stimulated A549 and 16HBE Cells

As demonstrated in the existing literature, IL-1β has been observed to induce inflammation in A549 and 16HBE cells (positive control) and dexamethasone has been shown to have anti-inflammatory effects on the same inflamed cells (control) [33,45]. The present study therefore analyzed the possible anti-inflammatory effects of probiotics on these cells.

CCL-2 is a proinflammatory cytokine that plays a pivotal role in the process of inflammation. It has been demonstrated to attract or enhance the expression of other inflammatory factors [46].

**Figure 2:**
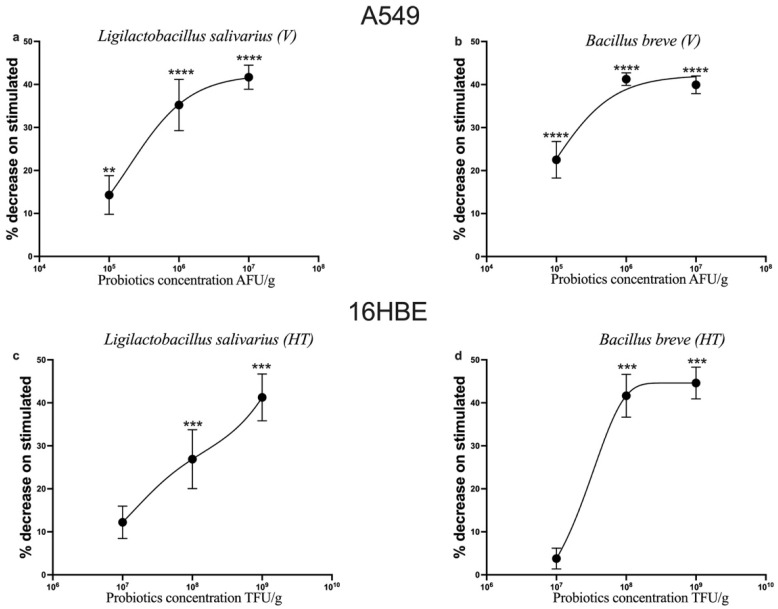
**Dose-dependent effects of probiotics on CCL-2 expression in IL-1β-stimulated epithelial cells.** A549 cells (**a**,**b**) and 16HBE cells (**c**,**d**). Cells were treated with increasing concentrations of *Ligilactobacillus salivarius*_V, *Bifidobacterium breve*_V, *L. salivarius*_HT and *B. breve*_HT, respectively (**a**–**d**), in the presence of IL-1β (10 ng/mL) for 24 h. Results are expressed as the percentage decrease relative to IL-1β-stimulated cells. Each value represents the mean ± S.E.M. (n = 5 for each panel). ** *p* < 0.01, *** *p* < 0.001, **** *p* < 0.0001 vs. IL-1β-stimulated cells (Student’s *t*-test).

Figure 2 shows the percentage of the decrease in CCL-2 expression in cells stimulated with IL-1β and treated with increasing concentrations of probiotics, in relation to the protein’s expression on cells only stimulated with IL-1β.

*L. salivarius_*V (Pb1) and *B. breve_*V (Pb3) showed a dose-dependent anti-inflammatory effect on the alveolar epithelial cell line (A549); instead, *L. salivarius_*HT (Pb2) and *B. breve_*HT (Pb4) showed a dose-dependent anti-inflammatory effect on bronchial epithelial cells (16HBE).

TNF-α is an inflammatory cytokine produced by macrophages/monocytes during acute inflammation. It is responsible for a diverse range of signaling events within cells, leading to necrosis or apoptosis. The protein is also important for resistance to infection and cancer [47].

**Figure 3:**
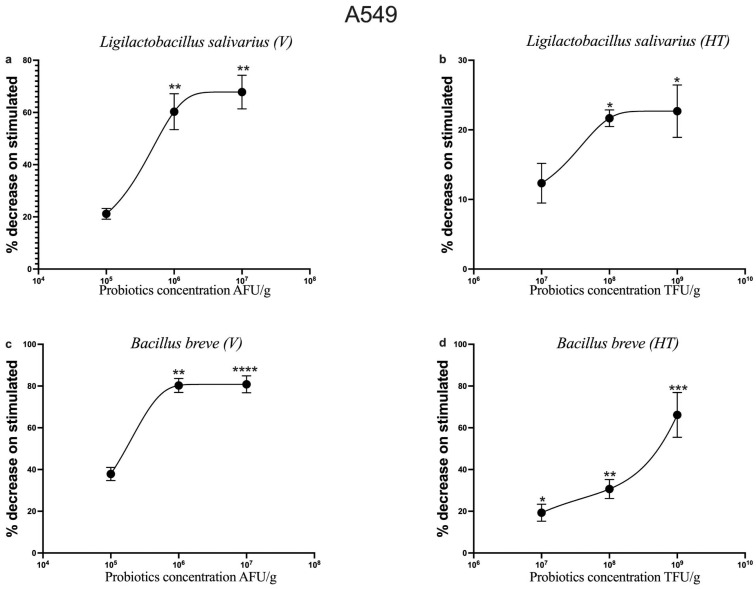
**Dose-dependent effects of probiotics on TNF-α expression in IL-1β-stimulated A549 cells**. A549 cells were treated with increasing concentrations of *Ligilactobacillus salivarius*_V, *L. salivarius*_HT (**a**,**b**), *Bifidobacterium breve*_V and *B. breve*_HT (**c**,**d**), respectively, in the presence of IL-1β (10 ng/mL) for 24 h. Results are expressed as the percentage decrease relative to IL-1β-stimulated cells. Each value represents the mean ± S.E.M. (n = 5 for each panel). * *p* < 0.05, ** *p* < 0.01, *** *p* < 0.001, **** *p* < 0.0001 vs. IL-1β-stimulated cells (Student’s *t*-test).

Figure 3 shows the percentage of the decrease in TNF-α expression on cells stimulated with IL-1β and treated with increasing concentrations of probiotics, in relation to the protein’s expression on cells only stimulated with IL-1β.

In contrast to CCL-2, all four probiotics exhibited a dose-dependent anti-inflammatory effect on the alveolar epithelial cell line (A549).

However, in the 16HBE cells, the lack of observations of even the controls precluded the assessment of the potential anti-inflammatory effect of probiotics.

### 3.3. Effects of Probiotics on IL-1β-Induced IL-4, Eotaxin, IL-6 and IL-8 Production in A549 and 16HBE Cell Lines

To further explore the anti-inflammatory potential of the tested probiotics, we evaluated their effects on a panel of cytokines, IL-4, eotaxin, IL-6 and IL-8, using their highest concentrations (10^7^ AFU/g for Pb1 and Pb3; 10^9^ TFU/g for Pb2 and Pb4). These cytokines were selected due to their established roles in the pathogenesis of airway inflammation and their responsiveness to IL-1β stimulation [37,40,48,49].

**Figure 4:**
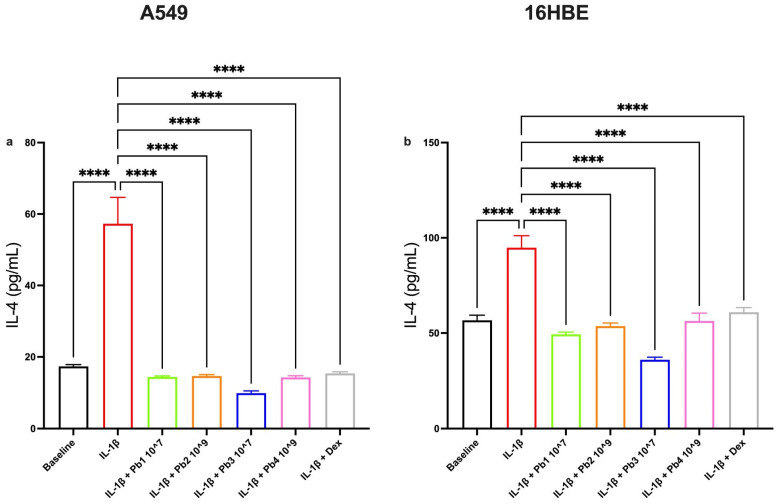
**Effects of Probiotics on IL-1β-induced IL-4 production in A549 and 16HBE cell lines**. Panel (**a**) shows IL-4 production in A549 cells under baseline conditions and following treatment with IL-1β alone or in combination with probiotics or dexamethasone (as control). Panel (**b**) shows IL-4 production in 16HBE cells under baseline conditions and following treatment with IL-1β alone or in combination with probiotics or dexamethasone (as control). All probiotics tested (*L. salivarius*_V: Pb1; *L. salivarius*_HT: Pb2; *B. breve*_V: Pb3; *B. breve*_HT: Pb4.) significantly attenuated IL-1β-induced IL-4 levels. Data are presented as the mean ± S.E.M. (n = 5 for each panel) Statistical significance was determined by one-way ANOVA followed by a post hoc test, **** *p* < 0.0001.

All four probiotics significantly reduced IL-4 levels in both A549 and 16HBE cells. This Th2-associated cytokine, which promotes eosinophilic inflammation and IgE switching [50], was consistently attenuated across both cell lines, suggesting a broad immunomodulatory capacity of both viable and heat-treated strains (Figure 4).

**Figure 5:**
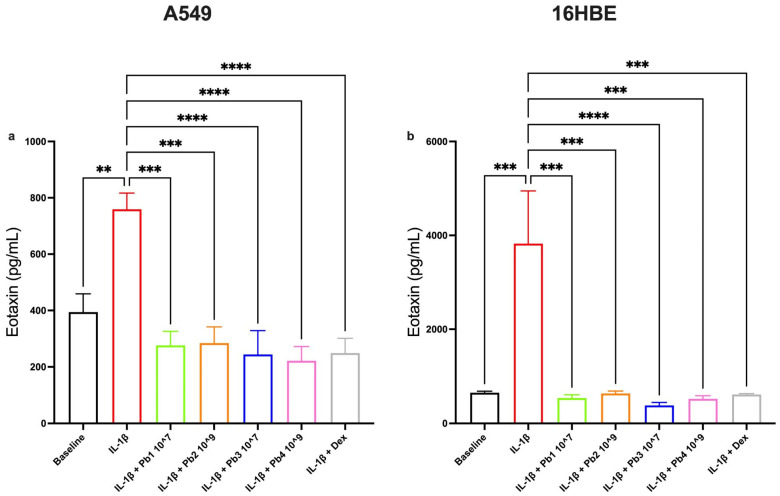
**Effects of Probiotics on IL-1β-induced Eotaxin production in A549 and 16HBE cell lines.** Panel (**a**) shows eotaxin production in A549 cells under baseline conditions and following treatment with IL-1β alone or in combination with probiotics or dexamethasone (as control). Panel (**b**) shows eotaxin production in 16HBE cells under baseline conditions and following treatment with IL-1β alone or in combination with probiotics or dexamethasone (as control). All probiotics tested (*L. salivarius*_V: Pb1; *L. salivarius*_HT: Pb2; *B. breve*_V: Pb3; *B. breve*_HT: Pb4.) significantly attenuated IL-1β-induced eotaxin levels. Data are presented as the mean ± S.E.M. (n = 5 for each panel). Statistical significance was determined by one-way ANOVA followed by a post hoc test. ** *p* < 0.01, *** *p* < 0.001, **** *p* < 0.0001.

Similarly, eotaxin production, a cystine-adjacent chemokine (C-C) involved in eosinophils, basophils and Th2 lymphocytes activation and often elevated in asthma [48], was also significantly reduced by all probiotic treatments in both cell lines. These findings indicate that both epithelial environments respond to probiotical modulation in a comparable manner when it comes to Th2-type chemotactic signals (Figure 5).

In contrast, the results for IL-6 and IL-8 were more cell-type specific. IL-6 is a pleiotropic cytokine involved in inflammation, immune response and hematopoiesis. Produced in response to infections and tissue injuries, it contributes to host defense by inducing acute phase proteins such as CRP and supports adaptive immunity by promoting antibody production and effector T-cells development. It can also drive the differentiation or proliferation of several non-immune cells [51].

IL-8, on the other hand, is a cytokine produced by phagocytes and mesenchymal cells exposed to inflammatory stimuli, such as IL-1 or TNF. It activates neutrophils by inducing chemotaxis, exocytosis and the respiratory burst. Along with related chemokines, IL-8 is released in various tissues in response to infection, inflammation, ischemia, trauma, etc., and it is thought that they are the main cause of local neutrophil accumulation [52].

**Figure 6:**
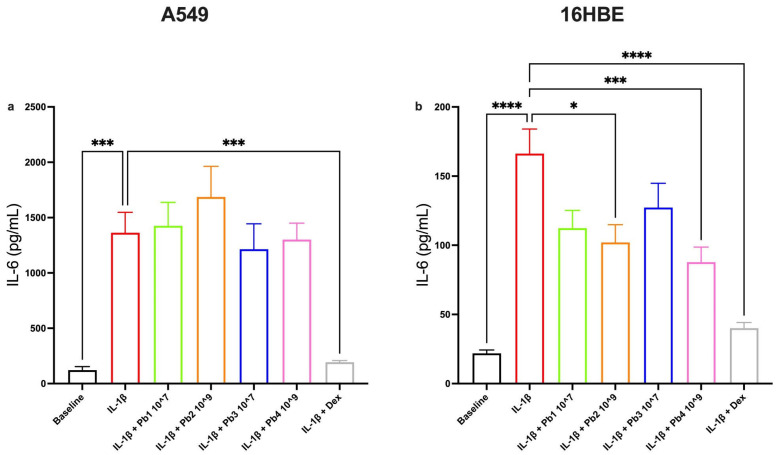
**Effects of Probiotics on IL-1β-induced IL-6 production in A549 and 16HBE cell lines.** Panel (**a**) shows IL-6 production in A549 cells under baseline conditions and following treatment with IL-1β alone or in combination with probiotics or dexamethasone (as control). The probiotics tested (*L. salivarius*_V: Pb1; *L. salivarius*_HT: Pb2; *B. breve*_V: Pb3; *B. breve*_HT: Pb4) did not significantly reduce IL-1β-induced IL-6 levels in A549 cells. Panel (**b**) shows IL-6 production in 16HBE cells under baseline conditions and following IL-1β alone or in combination with probiotics or dexamethasone (as control). Among probiotics tested, *L. salivarius*_HT: Pb2, showed moderate reduction (* *p* < 0.05), while *B. breve*_HT: Pb4, demonstrated stronger inhibition (*** *p* < 0.001) of IL-1β-induced IL-6 levels. Data are presented as the mean ± S.E.M (n = 5 for A549 cells, 8 for 16HBE cells). Statistical significance was determined by one-way ANOVA followed by a post hoc test, **** *p* < 0.0001.

**Figure 7:**
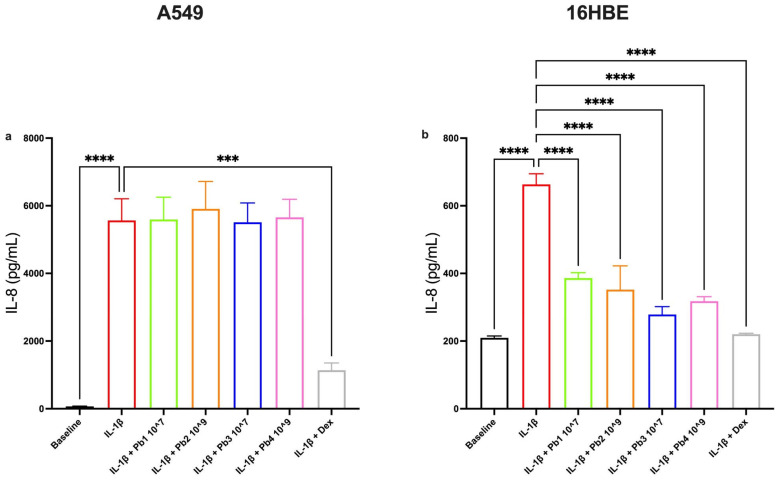
**Effects of Probiotics on IL-1β-induced IL-8 production in A549 and 16HBE cell lines.** Panel (**a**) shows IL-8 production in A549 cells under baseline conditions and following treatment with IL-1β alone or in combination with probiotics or dexamethasone (as control). The probiotics tested (*L. salivarius*_V: Pb1; *L. salivarius*_HT: Pb2; *B. breve*_V: Pb3; *B. breve*_HT: Pb4.) did not significantly reduce IL-1β-induced IL-8 levels in A549 cells. Panel (**b**) shows IL-8 production in 16HBE cells under baseline conditions and following treatment with IL-1β alone or in combination with probiotics or dexamethasone (as control). All probiotics tested (*L. salivarius*_V: Pb1; *L. salivarius*_HT: Pb2; *B. breve*_V: Pb3; *B. breve*_HT: Pb4) significantly attenuated IL-1β-induced IL-8 levels, **** *p* < 0.0001. Data are presented as the mean ± S.E.M (n = 5 for each panel). Statistical significance was determined by one-way ANOVA followed by a post hoc test, *** *p* < 0.001.

In A549 cells, none of the probiotic treatments led to a statistically significant reduction in IL-6 or IL-8 levels, despite the presence of robust IL-1β inducing upregulation and confirming the response to the dexamethasone control (Figure 6a and Figure 7a). However, in 16HBE cells, *L. salivarius*_HT (Pb2) and *B. breve*_HT (Pb4) significantly decreased IL-6 expression (Figure 6b), while all four probiotic strains significantly attenuated IL-8 levels (Figure 7b). This suggests that bronchial epithelial cells may be more responsive than alveolar cells to the probiotic modulation of these particular cytokines.

Together, these results highlight the capacity of both viable and heat-treated probiotics to downregulate key pro-inflammatory mediators, particularly those associated with eosinophilic inflammations and chemotaxis, while also revealing cell-line-specific differences in responsiveness for cytokines such as IL-6 and IL-8.

### 3.4. Effects of Probiotics on the NF-κB Pathway in Inflamed A549 and 16HBE Cells

In the context of the innate and adaptive immune responses, the NF-κB pathway plays a pivotal role in the activation of distinct signaling components, thereby regulating the release of cytokines. This protein complex is known to be highly activated at sites of inflammation, and it is considered to be a key regulator of the immune response to infection [44].

To explore the potential interaction between the four probiotics used and the NF-κB pathway, the expression of phosphorylated p65-NF-κB, the active transcriptional component, was measured.

**Figure 8:**
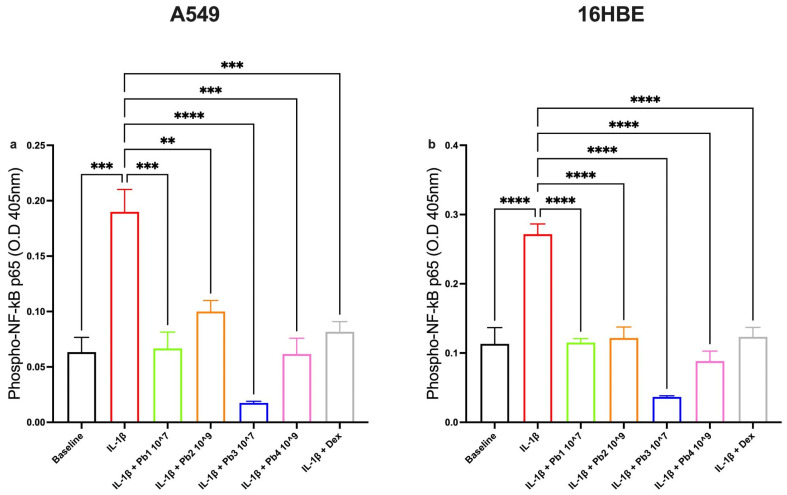
**Effects of Probiotics on IL-1β-induced NF-κB activation in A549 and 16HBE cell lines.** Panel (**a**) shows NF-κB activation in A549 cells under baseline conditions and following treatment with IL-1β alone or in combination with probiotics or dexamethasone (as control). IL-1β significantly increased NF-κB activation (*** *p* < 0.001), while all probiotics significantly reduced it (** *p* < 0.01, *** *p* < 0.001, **** *p* < 0.0001). In 16HBE cells, Panel (**b**), IL-1β significantly upregulated NF-κB activation (**** *p* < 0.0001), and all probiotics significantly attenuated this effect (**** *p* < 0.0001). Data are presented as the mean ± S.E.M (n = 3 for each panel). Statistical significance was determined by one-way ANOVA with a post hoc test.

Controls were validated for the two cell lines used. The results showed that all four probiotics had a significant anti-inflammatory effect on inflamed A549 and 16HBE cells (Figure 8). This effect was associated with a reduction in NF-κB activation, as the probiotics reduce the expression of phosphorylated p65-NF-κB. These findings suggest that the anti-inflammatory properties of the probiotics are closely linked to their ability to interfere with the NF-κB signaling pathway.

### 3.5. Effects of Probiotics on COX-2 and PGE2 Pathways in Inflamed A549 and 16HBE Cells

COX-2 and PGE2 are implicated in many normal cellular processes as well as pathophysiological processes such as inflammation, edema, bronchoconstriction, platelet aggregation, fever and hyperalgesia [44]; in general, they play a critical role in the pathogenesis of inflammatory diseases. In particular, the overproduction of PGE2 is observed in many of the human pathologies associated with inflammation and pro-tumoral conditions [42].

**Figure 9:**
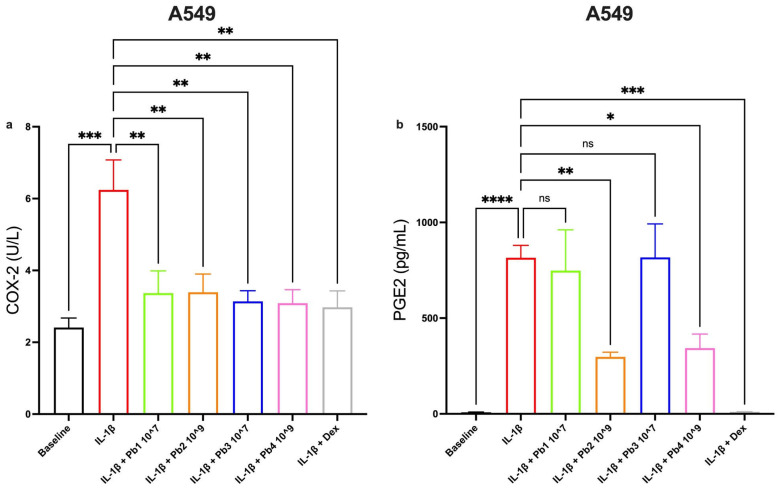
**Effects of Probiotics on IL-1β-induced COX-2 and PGE2 production in A549 cells.** Panel (**a**) shows that IL-1β significantly increased COX-2 (*** *p* < 0.001), with all probiotics significantly reducing this effect (** *p* < 0.01). PGE2 production (Panel (**b**)) shows IL-1β-induced upregulation (**** *p* < 0.0001) with variable probiotic effects; *L. salivarius*_HT: Pb2 and *B. breve*_HT: Pb4 showed significant reduction (** *p* < 0.01, * *p* < 0.05), while *L. salivarius*_V: Pb1 and *B. breve*_V: Pb3 had no significant effect. Data are presented as the mean ± S.E.M. (n = 6 for COX-2 experiments; 5 for PGE2 experiments). Statistical significance was determined by one-way ANOVA followed by a post hoc test. ns: not significant.

By focusing on the intracellular pathway, particularly the expression of COX-2, we can observe that, in addition to the controls, all probiotics are found to significantly reduce IL-1β activity on the A549 cell line (Figure 9a).

On the other hand, upon observing the expression of PGE2, in addition to the controls, only the heat-treated probiotics significantly reduce the expression of the protein studied (Figure 9b).

In 16HBE cells, the absence of observations of both controls prevented the evaluation of the potential anti-inflammatory effect of probiotics.

## 4. Discussion

The human respiratory tract is constantly exposed to environmental stimuli and potential pathogens, and its immune balance plays a crucial role in determining whether these exposures result in tolerance or inflammation. Emerging evidence suggests that the modulation of mucosal immunity through microbial-based intervention may offer novel strategies to prevent or ameliorate airway inflammatory disorders. However, their mechanisms of action remain incompletely understood, especially in the context of the lung epithelium [53]. In this study, we have evaluated the potential anti-inflammatory properties of two probiotic strains, *Ligilactobacillus salivarius* and *Bifidobacterium breve*, in both their viable and heat-treated (postbiotic) forms, using an in vitro model of lung inflammation based on A549 and 16HBE cell lines stimulated with IL-1β. A dose-response approach was employed to assess the effects of increasing concentrations of each preparation. Our results showed that all probiotic preparations were non-cytotoxic at the tested concentrations. Importantly, treatment with both viable and heat-treated probiotics led to a reduction in the expression of pro-inflammatory mediators, including CCL-2, TNF-α, IL-4, IL-6, IL-8 and eotaxin, although some cell-type-specific differences were noted. These cytokines are known to play critical roles in the recruitment of immune cells, mucus secretion and airway remodeling in inflammatory respiratory diseases such asthma and COPD [54,55]. The observed anti-inflammatory effects were closely associated with the downregulation of the NF-κB pathway, a central regulator of inflammatory responses. The phosphorylated form of the p65 subunit, a key indicator of NF-κB activation, was significantly reduced in cells treated with all probiotic forms. Our data also showed reduced expression of COX-2 and its downstream product PGE2, particularly in A549 cells, indicating interference with prostaglandin-mediated signaling, which is frequently implicated in airway hyperresponsiveness and inflammation [56]. Interestingly, the heat-treated forms of both strains were particularly effective in reducing PGE2 expression, highlighting the emerging therapeutic potential of postbiotics [57]. However, viable probiotics showed greater efficacy in modulating other cytokines, such as CCL-2 and TNF-α in A549 cells. This variability in activity between live and treated forms likely reflect differences in their model of action. Live probiotics can interact dynamically with host epithelial cells, producing bioactive metabolites in situ. In contrast, heat-treated strains exert their effects primarily through structural components, such as wall fragments, lipoteichoic acid or DNA, which can directly engage pattern recognition receptors, e.g., TLRs, on epithelial cells [58]. It is worth noting that the differential response between cell types, A549 vs. 16HBE, and between strains suggest that the anti-inflammatory properties of probiotics are not only strain-specific but also dependent on the epithelial context in which they act. These findings are in line with a growing body of evidence supporting the immunomodulatory activity of probiotics in airway epithelial models. For instance, Beak et al. [59] reported that heat-killed *Lactiplantibacillus plantarium* alleviated LPS-induced inflammation and apoptosis in A549 cells by downregulating pro-inflammatory cytokines such as IL-6 and eotaxin, inhibiting the NF-κB signaling pathways and reducing ROS generation. Similarly, Kang et al. [60] reported that heat-inactivated *Levilactobacillus brevis* exerts strong antioxidant and anti-proinflammatory effects in the same LPS-stimulated model, resulting in decreased production of IL-6, eotaxin and ROS. In addition, Kim et al. [61] showed that heat-killed *Lactilactobacillus sakei* reduced cytokines and chemokines gene expression inhibiting NF-κB activation in A549 cells exposed to LPS, supporting their anti-inflammatory and anti-asthmatic potential. Together with our results, these studies support the emerging concept that probiotics strains can modulate inflammation in airway epithelial cells. While our study offers important insights, it is not without limitations. The exclusive use of in vitro models does not fully capture the complexity of host–microbiome interaction in vivo, particularly in the presence of microbiota, immune cells and dynamic environmental exposure. Furthermore, the lack of consistent controls in some conditions, e.g., TNF-α and PGE2 in 16HBE cells, limited our ability to evaluate certain effects comprehensively. Future work will need to integrate more physiologically relevant systems, such as organoids or ex vivo tissue, to better validate the clinical potential of our results.

## 5. Conclusions

In conclusion, our study aimed to evaluate and compare the anti-inflammatory effects of viable and heat-treated probiotic strains in lung epithelial models, one of the first comprehensive efforts to do so. This objective was successfully achieved, as our results demonstrate that both *L. salivarius* and *B. breve* can effectively modulate pulmonary inflammation through the inhibition of NF-κB and COX-2 pathways. The implementation of flow cytometry-based enumeration enabled precise dose-response evaluations across a broad concentration range, providing robust evidence for the anti-inflammatory properties of both probiotic forms. Notably, our findings highlight the particular promise of postbiotic preparations for direct pulmonary applications. The heat-treated forms demonstrated substantial anti-inflammatory activity, particularly in reducing PGE_2_ expression, while eliminating concerns associated with administering viable microorganisms to the respiratory tract. This safety profile, combined with enhanced stability characteristics inherent to non-viable preparations, positions postbiotics as ideal candidates for incorporation into inhalation devices, nebulizers and other pulmonary delivery systems where maintaining microbial viability would be technically challenging or clinically inadvisable. The clinical relevance of these strains has been demonstrated in a recent randomized controlled trial [62], where oral administration of the same *L. salivarius* LS01 and *B. breve* BR03 combination significantly reduced respiratory tract infections in otherwise healthy children, with a 36% reduction in infection incidence and decreased severity of symptoms including cough and rhinorrhea. These clinical outcomes align remarkably with our in vitro findings of reduced inflammatory mediators, suggesting that the anti-inflammatory mechanisms we observed at the cellular level translate into meaningful clinical benefits. While the clinical study employed the traditional oral administration of viable probiotics, our data now suggest an innovative complementary approach: direct pulmonary delivery of the heat-treated forms could potentially enhance local anti-inflammatory effects in the respiratory tract while maintaining the systemic benefits observed with oral supplementation.

Overall, this study provides a solid foundation for future translational efforts and supports the relevance of in vitro models in bridging mechanistic findings and clinical applications. The strain-specific and form-dependent modulation of key inflammatory mediators observed in our model thus supports dual therapeutic strategies—both conventional oral probiotic supplementation and novel inhaled postbiotic formulations. This versatility underscores the therapeutic potential of these specific strains in respiratory health management. Future work should aim to confirm these findings in vivo and in clinical settings and to explore the potential of combined oral and inhaled approaches to maximize therapeutic outcomes.

## Data Availability

The datasets used and/or analyzed in the current study are available from the corresponding author on reasonable request.
